# Supplementary material for: Optimal switching between geocentric and egocentric strategies in navigation
Source: R Soc Open Sci. 2016 Jul 27;3(7):160128. doi: 10.1098/rsos.160128 (PMC4968461; doi:10.1098/rsos.160128)
Supplement: File: Supplementary Material Nav-Proc_Royal_Soc_B_Supp.pdf Description: Manipulation of the Lambert W-funciton. [file rsos160128supp1.zip › Nav-Proc_Royal_Soc_OS_Supp.pdf]

# Supplementary Material: **Optimal switching between geocentric and egocentric strategies for navigation**

O. Peleg[1] and L. Mahadevan[1, 2, *]

[1] *John A. Paulson School of Engineering and Applied Sciences, Harvard University, Cambridge, Massachusetts 02138, USA*

[2] *Departments of Physics, and Organismic and Evolutionary Biology, Wyss Institute and Kavli Institute, Harvard University, Cambridge, Massachusetts 02138, USA*

(Dated: June 18, 2016)

* lm@seas.harvard.edu

## I. MANIPULATION OF LAMBERT W-FUNCTION

Here we show that

$$\lim_{\theta^*\to 0} \frac{1+W\left(-\frac{\sin(\theta^*)}{e\theta^*}\right)}{-\log\left(\sin(\theta^*)/\theta^*\right)} = \frac{2\sqrt{3}}{\theta^*} + O(\theta^*), \tag{1}$$

where $W(z)$ is the Lambert W-function. First we note a few identities of the $W(z)$:

$$\frac{dW(z)}{dz} = \frac{W(z)}{z(W(z)+1)} \tag{2}$$

$$\lim_{z\to -\frac{1}{e}} W(z) \sim -1 + \sqrt{2e}\sqrt{z+\frac{1}{e}} + O(z). \tag{3}$$

Since $\lim_{\theta\to 0}\sin(\theta)/\theta \sim 1-\frac{\theta^2}{6}$, an expansion near $W(z\to -\frac{1}{e})$ is appropriate. Therefore, the numerator is:

$$\begin{aligned} 1+W\left(-\frac{\sin(\theta^*)}{e\theta^*}\right) &= 1-1+\sqrt{2e}\sqrt{\frac{1}{e}\frac{{\theta^*}^2}{6}} \\ &+O\left((\theta^*)^2\right) \\ &= \frac{\theta^*}{\sqrt{3}} + O\left((\theta^*)^2\right). \end{aligned} \tag{4}$$

Likewise, the expansion for the denominator is, $\log(\frac{\sin(\theta^*)}{\theta^*}) = -\frac{{\theta^*}^2}{6} + O\left({\theta^*}^4\right)$. Combining these, we demonstrate

$$\lim_{\theta^*\to 0} \frac{1+W\left(-\frac{\sin(\theta^*)}{e\theta^*}\right)}{-\log\left(\sin(\theta^*)/\theta^*\right)} = \frac{2\sqrt{3}}{\theta^*} + O(1). \tag{5}$$
