# Supplementary material for: Optimal switching between geocentric and egocentric strategies in navigation
Source: R Soc Open Sci. 2016 Jul 27;3(7):160128. doi: 10.1098/rsos.160128 (PMC4968461; doi:10.1098/rsos.160128)
Supplement: File: Supplementary Material Nav-Proc_Royal_Soc_B_Supp.pdf Description: Manipulation of the Lambert W-funciton. [file rsos160128supp1.zip › OPENSCIENCE.pdf]

ROYAL SOCIETY
OPEN SCIENCE
