# Supplementary figures and images for: Optimal switching between geocentric and egocentric strategies in navigation
Source: R Soc Open Sci. 2016 Jul 27;3(7):160128. doi: 10.1098/rsos.160128 (PMC4968461; doi:10.1098/rsos.160128)

A)

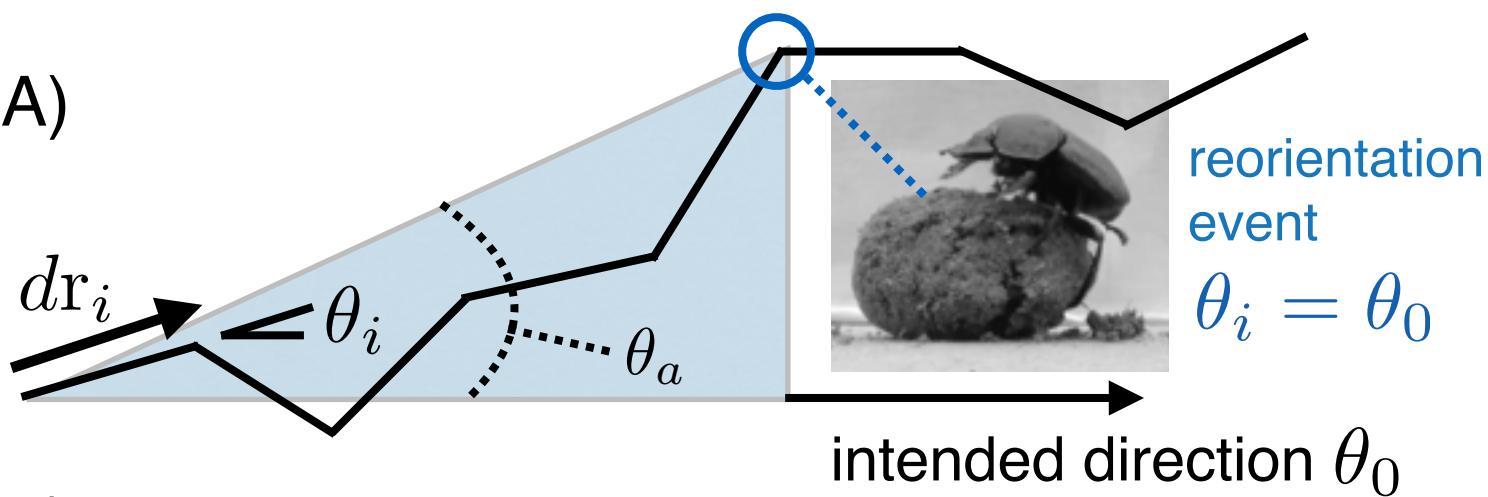

B)

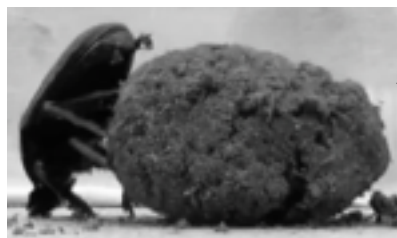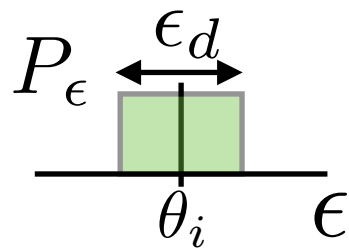**inattention**
 $(1 - \tau)N$   
steps
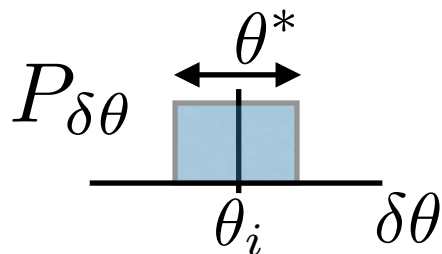**attention**
 $\tau N$   
steps
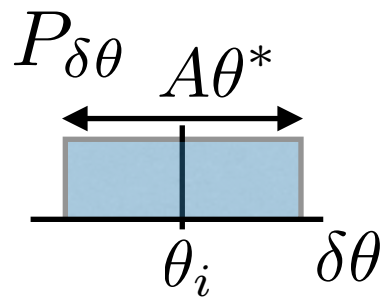

Supplement: File: Supplementary Material Nav-Proc_Royal_Soc_B_Supp.pdf Description: Manipulation of the Lambert W-funciton. [file rsos160128supp1.zip › fig1.pdf]

A)

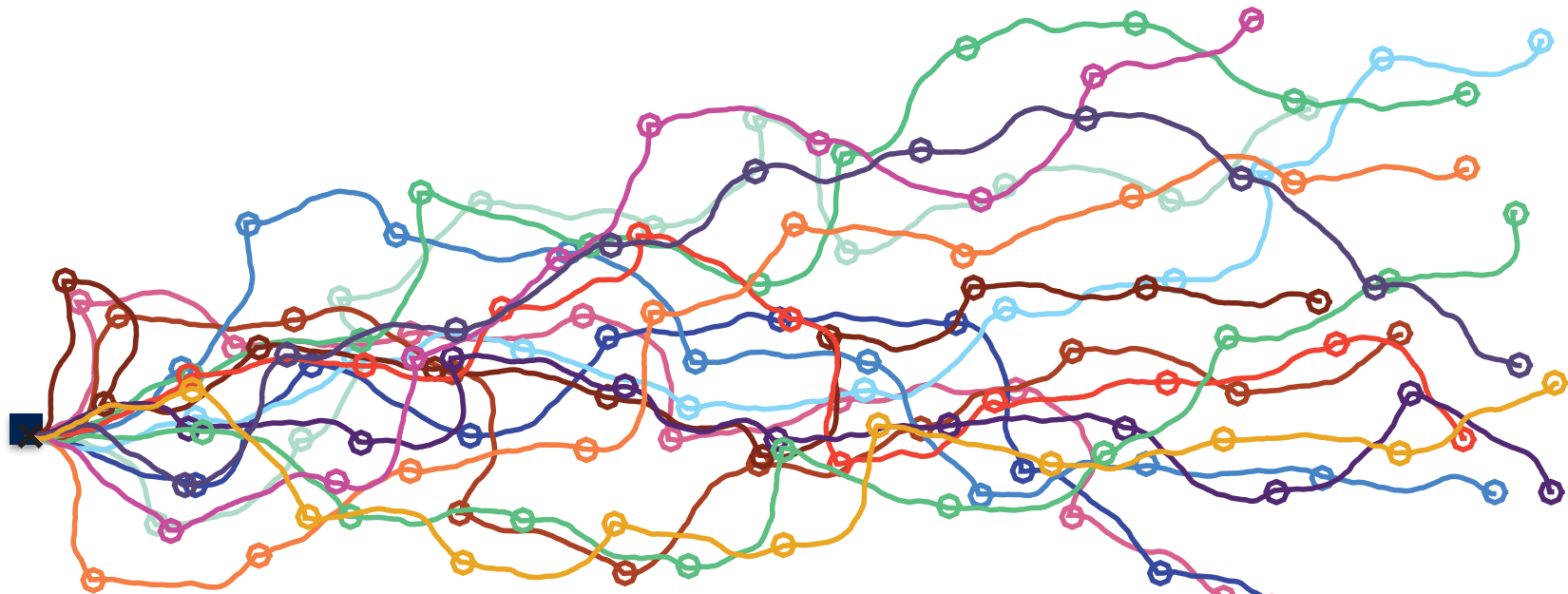

B)

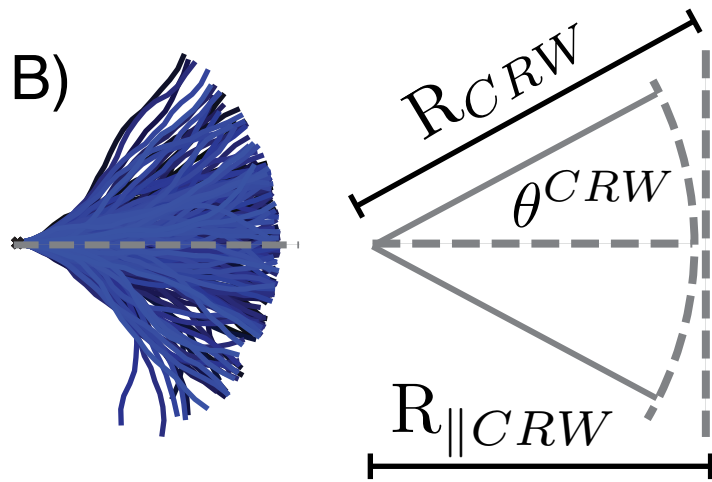

C)

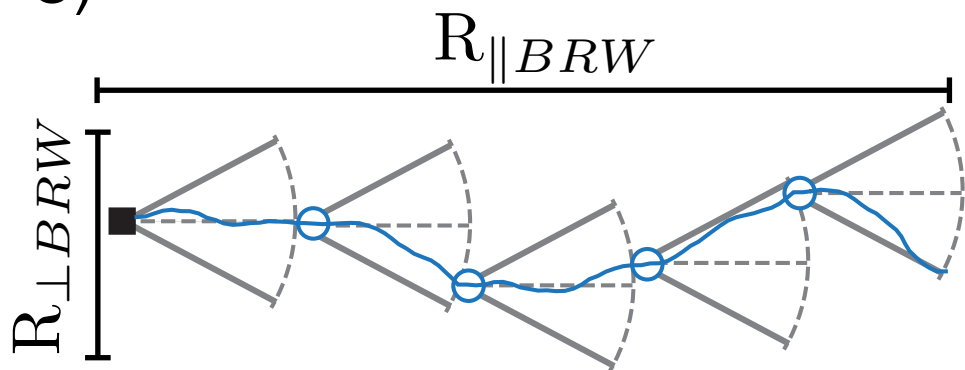

Supplement: File: Supplementary Material Nav-Proc_Royal_Soc_B_Supp.pdf Description: Manipulation of the Lambert W-funciton. [file rsos160128supp1.zip › fig2.pdf]

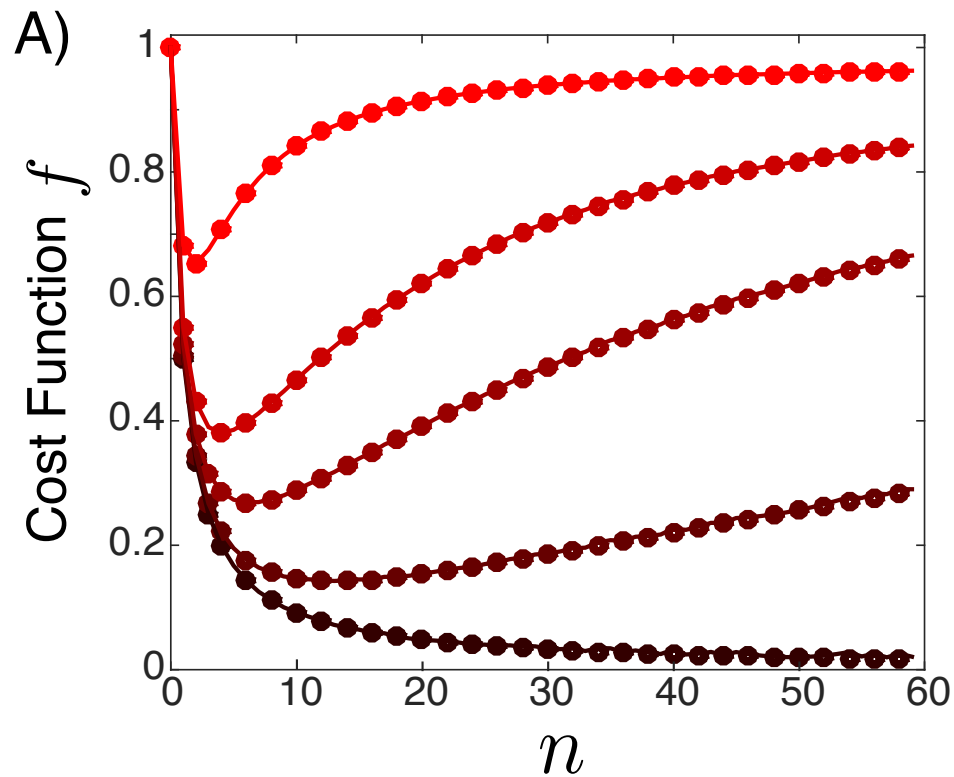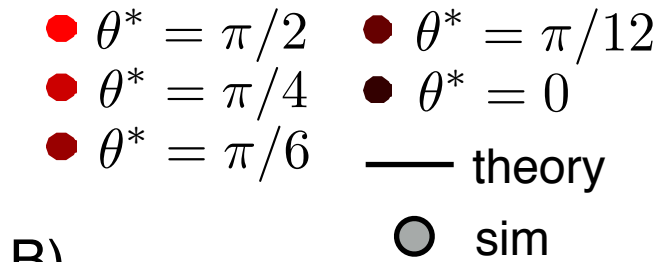

Supplement: File: Supplementary Material Nav-Proc_Royal_Soc_B_Supp.pdf Description: Manipulation of the Lambert W-funciton. [file rsos160128supp1.zip › fig3.pdf]

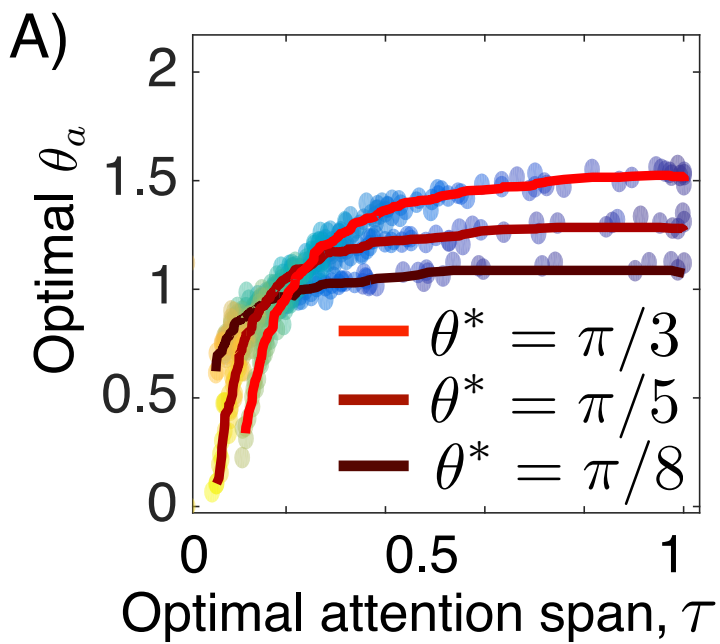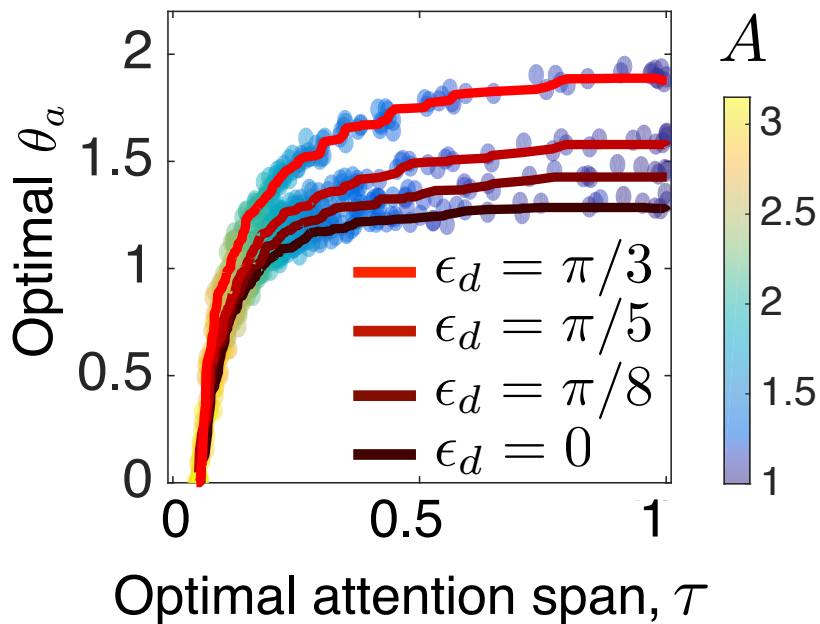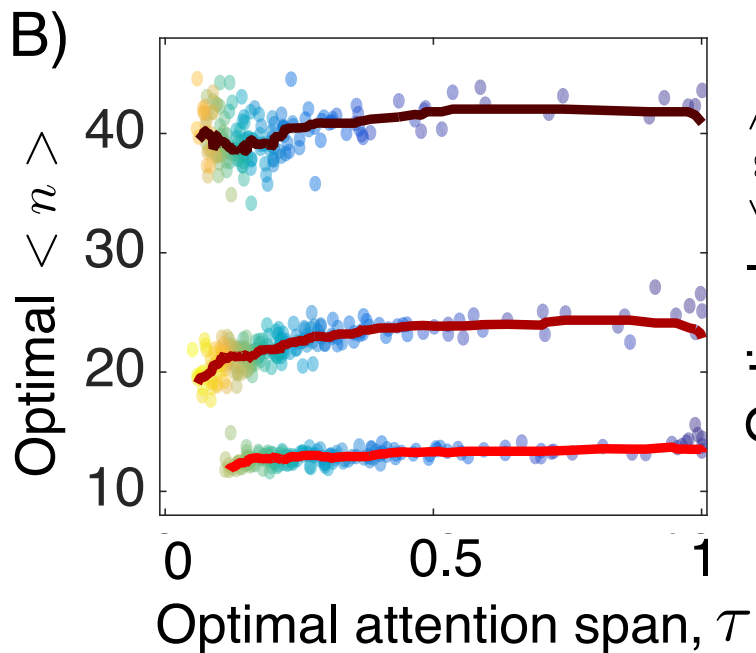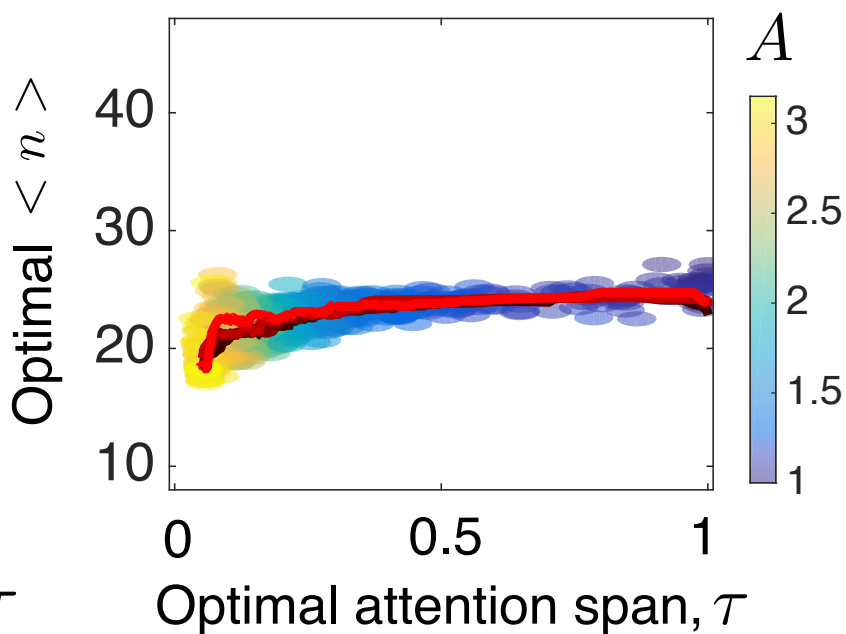

Supplement: File: Supplementary Material Nav-Proc_Royal_Soc_B_Supp.pdf Description: Manipulation of the Lambert W-funciton. [file rsos160128supp1.zip › fig4.pdf]

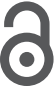

Supplement: File: Supplementary Material Nav-Proc_Royal_Soc_B_Supp.pdf Description: Manipulation of the Lambert W-funciton. [file rsos160128supp1.zip › openaccesslogo_bw.pdf]

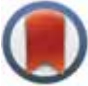

CrossMark

[click for updates](#)

Supplement: File: Supplementary Material Nav-Proc_Royal_Soc_B_Supp.pdf Description: Manipulation of the Lambert W-funciton. [file rsos160128supp1.zip › RS_crossmark_logo.pdf]

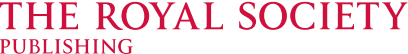

Supplement: File: Supplementary Material Nav-Proc_Royal_Soc_B_Supp.pdf Description: Manipulation of the Lambert W-funciton. [file rsos160128supp1.zip › RSOS_Pubs_Logo_Line_CMYK.pdf]
